# Modules in robust but low-efficiency phyllosphere fungal networks drive saponin accumulation in leaves of different *Panax* species

**DOI:** 10.1186/s40793-023-00516-7

**Published:** 2023-07-12

**Authors:** Guozhuang Zhang, Liping Shi, Congsheng Liu, Renjun Mao, Bing Xia, Zhixin Huang, Xiuye Wei, Lixuan Wu, Yuqing Zheng, Guangfei Wei, Jia Xu, Shuangrong Gao, Shilin Chen, Linlin Dong

**Affiliations:** 1 Key Laboratory of Beijing for Identification and Safety Evaluation of Chinese Medicine, Institute of Chinese Materia Medica, China Academy of Chinese Medical Sciences, Beijing, 100700 China; 2 Zhangzhou Pien Tze Huang Pharmaceutical Co., Ltd., Fujian, 363000 China; 3 School of Life Sciences, Yan’ an University, Yan’ an, 716000 China; 4 Institute of Herbgenomics, Chengdu University of Traditional Chinese Medicine, Chengdu, Sichuan China

**Keywords:** Microbial ecological network, Network robustness, Community assembly, Plant-fungi interaction, Plant secondary metabolite

## Abstract

**Background:**

The phyllosphere mycobiome plays a crucial role in plant fitness and ecosystem functions. The complex microbial ecological networks (MEN) formed by these fungi remain poorly understood, particularly with regard to their organization strategy and their contributions to plant secondary metabolites such as saponin.

**Results:**

In this study, we constructed six MENs from leaf epiphytic and endophytic mycobiomes of three *Panax* species distributed in the northeast and southwest ends of mainland China. Hub nodes were absent in these MENs, which were significantly more complex, robust, and less efficient compared to random graphs (*P* < 0.05), indicating a hub-independent high-robustness strategy to maintain structural homeostasis. The important roles of specific MEN modules in shaping leaf saponin profiles of each *Panax* species were proved by multiple machine learning algorithms. Positive regulation modules (PRMs) of total saponin content were further identified, which exhibited more deterministic ecological assembly and comprised of highly connected nodes as well as higher proportion of plant-associated fungal guilds compared to other network members, indicating their tight links with host plant. The significant and direct effects (*P* < 0.05) of PRMs on total saponin accumulation were validated by well-fitted structural equation models (χ^2^ < 0.3, *P* > 0.5). Taxonomic analysis revealed that Pleosporales and Chaetothyriales were significantly overrepresented by positive regulation taxa (PRT) of total saponin content (FDR < 0.05). Across PRT identified in three *Panax* species, *Epicoccum* and *Coniothyrium* were conservatively present, while species-specific taxa such as Agaricales were also found, indicating the conservatism and specificity of plant-fungi interactions associated with leaf saponin accumulation in *Panax* genus.

**Conclusions:**

These findings provide a foundation for understanding mechanisms maintaining the steady state of phyllosphere mycobiome in healthy plant, and offer clues for engineering phyllosphere mycobiome to improve the accumulation of bioactive secondary metabolites on the basis of network modules.

**Supplementary Information:**

The online version contains supplementary material available at 10.1186/s40793-023-00516-7.

## Background

The phyllosphere is the aerial parts of plants, and accounts for approximately 60% of the total biomass on Earth, providing habitats for diverse microorganisms such as bacteria and fungi [5, 71]. Among microbial lives in phyllosphere, leaf-associated microbiomes, including those residing on leaf surface (i.e., epiphytes) or in leaf tissues (i.e., endophytes), plays crucial roles in plant health, growth, and biogeochemical cycles [58]. While leaf bacterial communities have been extensively studied in term of mechanisms underlying community assembly and homeostasis, as well as their effects on plant immune response and carbon emission [1, 13, 44], knowledge about leaf mycobiomes is still lacking, particularly with regard to the maintenance strategy of community structure and their contributions to plant secondary metabolism [58, 75]. This knowledge is important for a more comprehensive understanding of phyllosphere biosphere and its role in plant health and ecology.

Intensive exchanges of substances, energy, and information among microorganisms in the phyllosphere shape complex interspecies interactions that can be represented by microbial ecological networks (MEN) [81]. Revealing the organization strategy of MEN can help us understand the potential mechanisms underlying the maintenance of community homeostasis [74]. Two strategies may be employed by microbial communities at the MEN level to adapt to phyllosphere of healthy plant and maintain their structure. The first strategy is high-efficiency. Efficiency is an important property measuring how fast information spreads across network [42]. A high-efficiency network relies on hub nodes which can shorten path length between any nodes, enabling external perturbation to distribute rapidly throughout the entire graph [32, 74]. Regarding the highly metabolic plasticity of fungi, the fast perception of disturbance such as species loss by fungi may allow them change gene expression profiles to adapt to abiotic or biotic changes timely [20, 54, 64]. A dynamic homeostasis can thus be achieved by high-efficiency fungal MENs. The second strategy is high-robustness, which maintains network structure by avoiding the spread of perturbation (e.g., node loss) across network. For instance, nodes can group separately, isolating the adverse effects caused by perturbation in a local area. The high-robustness network may also abandon hub nodes to prevent the destructive effects of hub loss on network structure, at the cost of reducing network efficiency [9]. It is unclear which strategy is preferred by phyllosphere mycobiomes. Given the harsh habitat of the phyllosphere with limited nutrients which may be insufficient to support efficient communication and rapid metabolic response of fungi, we hypothesize that phyllosphere fungal networks apply the hub-independent high-robustness strategy to organize themselves and maintain structure homeostasis (***H1***).

Phyllosphere microorganisms have close interactions with host plants [6], [58]. However, our knowledge of the effects of phyllosphere fungi on plant secondary metabolites remains limited. In the rhizosphere and root compartments, researches have shown the significant effects of microbiomes on the accumulation of plant secondary metabolites, especially saponins such as ginsenoside and glycyrrhizin [38, 66], [78]. Saponins are inducible phytoanticipins that play a crucial role in plant defense against bio-invasion, and the biosynthesis of which can be triggered by plant immune responses [23, 52]. As the plant immune response is also effective in the phyllosphere, we expect that certain members of the phyllosphere mycobiome can promote the accumulation of specific and/or total saponin in leaves. MENs typically exhibit modular structure in which nodes are closely connected by sharing environmental or resource preferences [18]. Network modules are thought to represent important ecological units, which may have significant implications for biological or ecological functions [37, 55, 67]. We thus hypothesis that certain MEN modules in the phyllosphere contribute to the formation of saponin profiles and the accumulation of total saponin in plant leaves (***H2***).

In this study, we aimed to test the above two hypotheses using a total of 162 fungal communities collected from the leaf endosphere (LE) and phylloplane (LP) of three *Panax* species: *P. ginseng* (PG), *P. quinquefolium* (PQ), and *P. notoginseng* (PN) [78]. Although *Panax* genus consists of about 20 species, the three species chosen in this study are especially famous for their wide consumption as dietary supplements, functional food, and medicinal materials for thousands of years, thus becoming the representative and best-selling ginseng species [28]. The three *Panax* species are closely related but distributed distantly, containing diverse ginsenosides as the major bioactive secondary metabolites [28]. By exploring the effects of phyllosphere fungi on saponin metabolism, we hope to improve the production of these pharmacologically important saponins through utilizing foliar microorganisms. This type of exploration has been carried out in several plant species [62]. For instance, the inoculation of *Epichloe festucae* raised the expression level of phenylpropanoid biosynthetic pathway in ryegrass, favoring the biosynthesis of secondary metabolites like flavonoids and anthocyanins [21]. Aly et al. [2] proposed the potential of fungal endophyte acting as biological trigger to increase the production of specific secondary metabolites of host plant. Combined with metadata including the contents of diverse types of saponins in leaves and edaphic properties, this study aims to: (i) estimate the strategies employed by fungal MENs to maintain community homeostasis, i.e., hub-dependent high-efficiency strategy or hub-independent high-robustness strategy,(ii) identify modules in MENs and their links to saponin profiles and total saponin accumulation in *Panax* leaves; and (iii) explore the relationships among taxa that potentially promoting saponin accumulation across the three *Panax* species to gain a better understanding of the evolutionary characteristics of plant-fungi interactions associated with plant chemical response.

## Methods

### Collection of samples and metadata

The sample collection has been detailly described in a previous study [78]. Specifically, *P. ginseng* (PG) and *P. quinquefolium* (PQ) plants were sampled from Jilin Province in the middle of September of 2019, with the mean annual temperature (MAT) ranging from 2.9 to 3.2 °C. While *P. notoginseng* (PN) plants were collected from Yunnan Province in late October of 2019, with the MAT ranging from 16.3 to 16.7 °C (Additional file 1: Fig. S1a). All plants were planted in fields through seed rather than transplanting, and all fields were managed following the Good Agricultural Practice [76], [79]. For each *Panax* species, ninety healthy plants were collected from each of three plantations cultivated with 2-year-old, 3-year-old, and 4-year-old plants, respectively. In each plantation, ten plants representing one sample were sampled from one of nine 2.0 × 1.5 m^2^ plots. A total of 27 samples representing 270 plants were obtained for each *Panax* species.

Fungi residing on leaf surface (LP) were collected according to Yao et al. [72]. Simply, plant leaves were placed into sterile 50-ml centrifuge tubes and immersed in sterile ultrapure water. After alternating sonication and vortex, the suspensions were centrifuged at 10,000 × g for 10 min, and the sediments were collected for DNA extraction and LP mycobiome profiling. After surface sterilized, the treated leaves were stored at − 80 °C for profiling of mycobiome in leaf endosphere (LE) and saponin quantification. Additionally, the bulk soil samples (BS) were collected from each plot for measuring edaphic factors using standard methods, including pH, organic carbon (OC), total nitrogen (TN), nitrate and ammonium nitrogen (NIN & AMN), available phosphorus (AP), available potassium (AK), exchangeable calcium (ECa) and magnesium (EMg), available sulfur (AS), and moisture content (MC) (Zhang et al., 2022). As the mean annual temperature (MAT) is similar among three plantations of each plant species, we did not account for temperature in subsequent analysis (Additional file 1: Fig. S1a). In addition, the soil MC rather than mean annual precipitation was used for estimating water conditions of plant, as all plants were cultivated in sunshade (Additional file 1: Fig. S1b).

For saponin quantification, 0.2 g well-mixed dried powder of leaves was weighed accurately and extracted using 15 ml pure methanol. After centrifuging and filtrating, the supernatant was injected into HPLC system (Waters Corporation., Milford, MA, USA). The elution gradient was set as follows: 0–12 min, 19% Acetonitrile (A) and 81% ultrapure water (B); 12–70 min, 19–36% A; 70–71 min, 36–19% A; 71–76 min, 19%A (Zhang et al., 2022). The retention times and contents of major saponins, including ginsenosides Rb1, Rb2, Rc, Rd, Re, Rg1, and F1 and notoginsenosides R1, were determined using corresponding standard substances (Shanghai Yuanye Bio-Technology Co., Ltd., Shanghai, China).

### DNA extraction and amplicon analysis

Total DNA was extracted from phylloplane precipitates and leaf tissues using FastDNA SPIN Kit for soil (MoBio Laboratories, Inc., Carlsbad, CA, USA). The ITS1F/ITS2R primer pairs were used to amplify fungal sequences, which were then sequenced by Illumina MiSeq PE 300 platform (Shanghai Biozeron Co., Ltd., Shanghai, China). Low quality reads and adaptors in raw sequences were removed using the fastp software (default parameters) [12]. The clean data were then submitted to the cutadapt software to remove the primes with the following parameters: “–errors 0.13 || –overlap 5” [45]. The obtained sequences were imported to QIIME2 using the import plugin [8]. The dada2 denoise-paired command was applied to denoise the sequences to generate amplicon sequence variants with the following parameters: “–p-trunc-len-f 220 || –p-trunc-len-r 220” [11]. The classify-sklearn method in QIIME2 was applied to perform taxonomy annotation using bayes-trained classifier on the basis of UNITE database (V16.10.2022) with the default parameter [48]. Unclassified and singleton ASVs were removed from our data, and remaining ASVs were inspected for plant DNA contamination using blast analysis. The obtained ASV table was then resampled to 12,412 sequences per sample to eliminate the effect of sequence depth difference.

### Statistical analysis

All statistical analyses were conducted in *R* (v4.2.2). *R* codes used for statistical analyses are available at https://github.com/githubzgz. All *P* values in multiple comparisons were adjusted according to Benjamini and Hochberg [7], and the adjusted *P* values were displayed as FDR.

#### MENs construction

Fungal MENs were constructed based on Spearman correlations among ASVs, following a random matrix theory (RMT)-based method to determine the correlation cut-off threshold in a non-arbitrary way [74]. This strategy has been widely applied in microbial ecology researches, greatly promoting our understanding of interaction structure, environmental response, and homeostasis maintenance of microbial communities in diverse habitats [22, 55, 70, 80]. Simply, the RMT-based method estimates nonrandom properties of complex systems like fungal communities from random noise, using the nearest-neighbor spacing distribution of eigenvalues [19], [82]. The transition of eigenvector distribution from GOE (Gaussian orthogonal ensemble) to Poisson distributions is typically used as a reference point to identify system-specific and nonrandom properties embedded in high-dimensional community data. The RMT-based method keeps us away from arbitrary selection of correlation cut-off, which is commonly used in microbial network studies. In the present study, fungal ASVs with relative abundances higher than 0.01% were selected to calculate Spearman correlations to reduce spurious associations caused by rare taxa [31]. After removing non-significant correlations (FDR > 0.05), the eigenvalue distribution of correlation matrix was analysed using the RMThreshold package to determine the transition point [39]. The matrix was then filtered based on transition point and the igraph package was used to construct network [16]. A total of six networks were constructed (three plant species × two compartments) (Additional file 1: Table S1).

#### Drivers of MENs

In addition to biotic interaction, environmental filtering and dispersal limitation are also considered as drivers of species co-occurrence or co-exclusion in MENs [36]. A recently developed method, named LTED (Link Test for Environmental filtering or Dispersal limitation), was applied to estimate the potential contributions of environment and space factors to observed links in MENs [68], [74]. In brief, if a link between two ASVs (i.e., Spearman correlations stronger than transition point) results from their covariations with one or more environmental factors, it is considered to be indirect and represent the influence of environmental filtering. For strength of dispersal limitation, all nodes in MENs were tested for their Mantel correlations with spatial distance based on Bray–Curtis dissimilarities [74]. If two linked nodes were both strongly affected by dispersal limitation (*r* ≥ 0.6, *P* < 0.05), their link is considered to result from dispersal limitation. Links were identified as the result of potential bio-interactions if they did not meet the above criteria.

#### MEN efficiency and robustness

To estimate the organization strategy employed by fungal MENs to maintain homeostasis, we estimated a series of topological properties for observed MENs and random graphs which had the same number of nodes and links with the observed graph. Specifically, the average shortest path length (avgL) and global efficiency were calculated to represent network efficiency. Smaller avgL and higher global efficiency indicated a potentially more efficient network. The natural connectivity was calculated for complete graphs and graphs with 20%, 30%, and 40% of nodes being randomly removed to measure structural robustness [4], [33]. Mean value and standard deviation of the above topological properties were calculated according to 100 random graphs generated based on Erdös-Réyni model using the sample_gnm function in the igraph package [56]. Deviations greater than 1.96 standard deviations from mean value of random graphs were considered as significant differences between observed and random graphs (*P* < 0.05). Other network properties, including degree centralization, clustering coefficient, and modularity (fast greedy algorithm), were also calculated for true and random graphs to estimate network complexity [41], [56]. To estimate whether fungal MENs were dependent on hub nodes, the *z*_*i*_ (within module degree) and *P*_*i*_ (participation coefficient) scores were calculated for each node according to Guimera and Nunes Amaral [26]. Nodes were then categorized as kinless hubs (*z*_*i*_ > 2.5, *P*_*i*_ > 0.62), provincial hubs (*z*_*i*_ > 2.5, *P*_*i*_ ≤ 0.62), connectors (*z*_*i*_ ≤ 2.5, *P*_*i*_ > 0.62), or peripherals (*z*_*i*_ ≤ 2.5, *P*_*i*_ ≤ 0.62) (Shi et al., 2020). Kinless and provincial hubs were considered as hub nodes.

#### Contribution of modules to leaf saponin profiles

For each *Panax* species, the differences of saponin profiles in leaves were represented by the first principal components (PC1), as they accounted for the major variations in contents of various saponins (PG: 59.07%; PQ: 73.91%; PN: 94.53%). The principal components analysis (PCA) of saponins were conducted using the rda function in the vegan package [50]. As the saponin contents were obtained using the absolute quantification method based on standard substances rather the relative ion intensity, we did not perform scale transformation in this analysis. Modules in MENs were detected using the cluster_fast_greedy function in the igraph package [15]. The fast greedy algorithm has been widely used in MEN analysis [19, 42]. Module eigengene was calculated for modules with 5 or more nodes in all fungal MENs, representing the variations in relative abundance of all ASVs within certain module [82]. Spearman correlation analysis was firstly performed between module eigengenes and saponin variations to identify candidate modules (FDR < 0.05) that potentially drove accumulation of different types of saponins. Candidate environmental factors were also selected using the same method. Before the analysis, environmental variables besides soil pH were log-transformed according to needs to improve normality, and variables with strong collinearity were removed (Spearman *ρ*^2^ > 0.6) [29]. As our previous study has shown the minor role of plant growth year in shaping mycobiome structures and saponin variations [78], and *Panax* leaves fell and regrew annually, we did not account for growth year here. To estimate that whether the incorporation of candidate fungal modules could improve our ability to predict saponin profiles, a machine learning framework was applied [51]. Simply, two machine learning algorithms were used for mutual authentication, including random forest analysis and *K*-nearest neighbors regression. Model performances were evaluated by mean squared error (MSE) and Spearman *ρ* between true observed response and predicted response. Lower MSE or higher Spearman *ρ* indicated a better model. Performance measures were calculated based on fivefold resampling, and the aggregated scores were extracted. Four models were fitted for each machine learning algorithm, in which saponin PC1 was used as response, and candidate environmental factors (Env), Env and candidate module eigengenes in LE networks (EnvLE), Env and candidate module eigengenes in LP networks (EnvLP), as well as Env and all candidate module eigengenes (EnvALL) were used as features, respectively. Machine learning-based analysis was conducted using the mlr3 package [34]. The as_task_regr function was used to construct machine learning tasks. The learners, resampling strategies, and model evaluator were created using the lrn, rsmp, and msrs functions, respectively. The resample function was applied to conduct the resampling procedures.

#### Identification of positive regulation modules and nodes

In modules significantly correlated to saponin variations (i.e., the candidate modules), those containing half of the number of nodes that exhibited significant and positive links to total saponin content (i.e., the sum of different types of saponins) were identified as positive regulation modules (PRMs) of total saponin. Node degree were compared between PRMs and other nodes in the same network to estimate the topological importance of PRM members. For the comparison of functional composition between PRMs and other nodes in the same network, we predicted the potentially functional guilds of MEN nodes using FUNGuild [47]. Only probable and highly probable predictions were retained in this analysis [18]. The community assembly processes of subcommunities comprising of PRM members and other nodes were examined using a taxonomic null model called Normalized Stochasticity Ratio (NST), respectively [49]. The NST measures the relative contribution of stochastic processes (e.g., dispersal process and ecological drift) to community assembly, which was calculated using the tNST package [49, 59]. A lower NST value indicates that the community is assembled by more deterministic processes such as selection [49, 59].

A series of structural equation modules (SEMs) were then fitted to test the direct effects of PRMs on total saponin accumulation. The candidate environmental variables and PRM eigengenes were incorporated into SEMs. The fit-of-goodness was estimated based on χ^2^ test, comparative fit index (CFI), and root mean square error of approximation (RMSEA). The SEM analysis was performed using the lavaan package [53]. In SEM-validated PRMs, fungal nodes significantly and positively correlated to total saponin content were identified as positive regulation taxa (PRT). An order-level enrichment analysis was performed to assess which order was significantly overrepresented by PRT using the phyper function in stats package. Simply, the ratio of PRT in each fungal order (i.e., the number of PRT in each fungal order/the number of all ASVs in MENs belonging to that order) was compared with the ratio of that order in MENs (i.e., the number of all ASVs in MENs belonging to that order / the number of all ASVs in MENs). Fungal phylogenetic tree was constructed based on taxonomic information using the taxonomy_to_tree.perl script developed by Tedersoo et al. [61]. The specificity and conservatism of these PRT across three *Panax* species were further visualized based on the tree using the ggtree package [73].

## Results

### Potential bio-interactions were main driver of links in fungal MENs

**Fig. 1:**
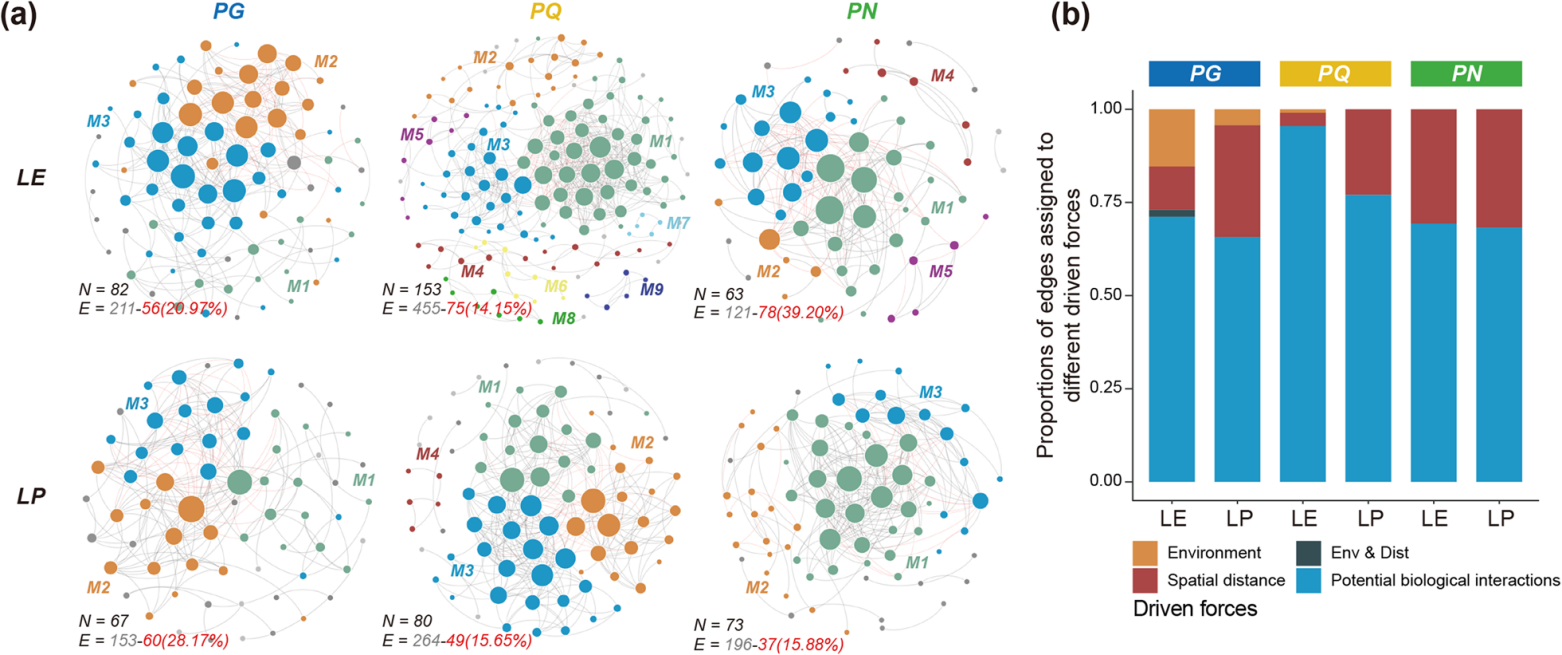
Fungal MENs and the relative contribution of different drivers to network links. **a** Six fungal networks constructed for two compartments of three *Panax* species. Node colors represent modules with 5 or more nodes. Gray and red links represent positive and negative correlations stronger than threshold, respectively. Digits displayed on left bottom of networks represent the number of nodes, positive and negative links, respectively; **b** The proportion of edges assigned to different driven forces calculated according to LTED. Environment represents edges which can be attributed to covariations of ASVs to environment variables. Spatial distance represents edges that can be attributed to dispersal limitation. Env & Dist represents edges driven by both environmental filtering and dispersal limitation. Remaining edges are assigned to the force of potential biological interactions

A total of six networks were constructed for three plant species and two compartments, with node number ranging from 63 to 153 and link number ranging from 199 to 530 (Fig. 1a**,** Additional file 1: Table S1). All MENs were dominated by positive links, which accounted for 60.80–85.35% of all edges (Fig. 1a). The distribution of node degree in all MENs followed a power law (Kolmogorov–Smirnov test, *P* > 0.05) in upper tail of degree higher than median, indicating the weakest scale-free according to the classification system developed by Broido and Clauset [10]. In which, only the network recovered from the LE of PQ exhibited higher than 50 nodes covered by power law and could be categorized as weak scale-free (Additional file 1: Fig. S2). Compared to phylloplane networks, the endosphere MENs exhibited lower graph density in PG and PQ, while the opposite result was observed in PN (Additional file 1: Table S1).

The potential drivers of network links were further estimated by LTED. In all MENs, only 4.53% to 34.27% of all edges could be assigned to environmental filtering and/or dispersal limitations, implying the dominated role of biological interactions in shaping networks (Fig. 1b). However, given the limitations of the environmental factors we measured and the nature of the correlation-based network approach, we still treated the links in MENs as putative bio-interactions. In summary, these results indicated that fungal MENs in *Panax* phyllosphere were weak scale-free networks, in which fungal interactions potentially contributed the most to observed links.

### Networks in *Panax* phyllosphere were robust but low-efficiency

**Fig. 2:**
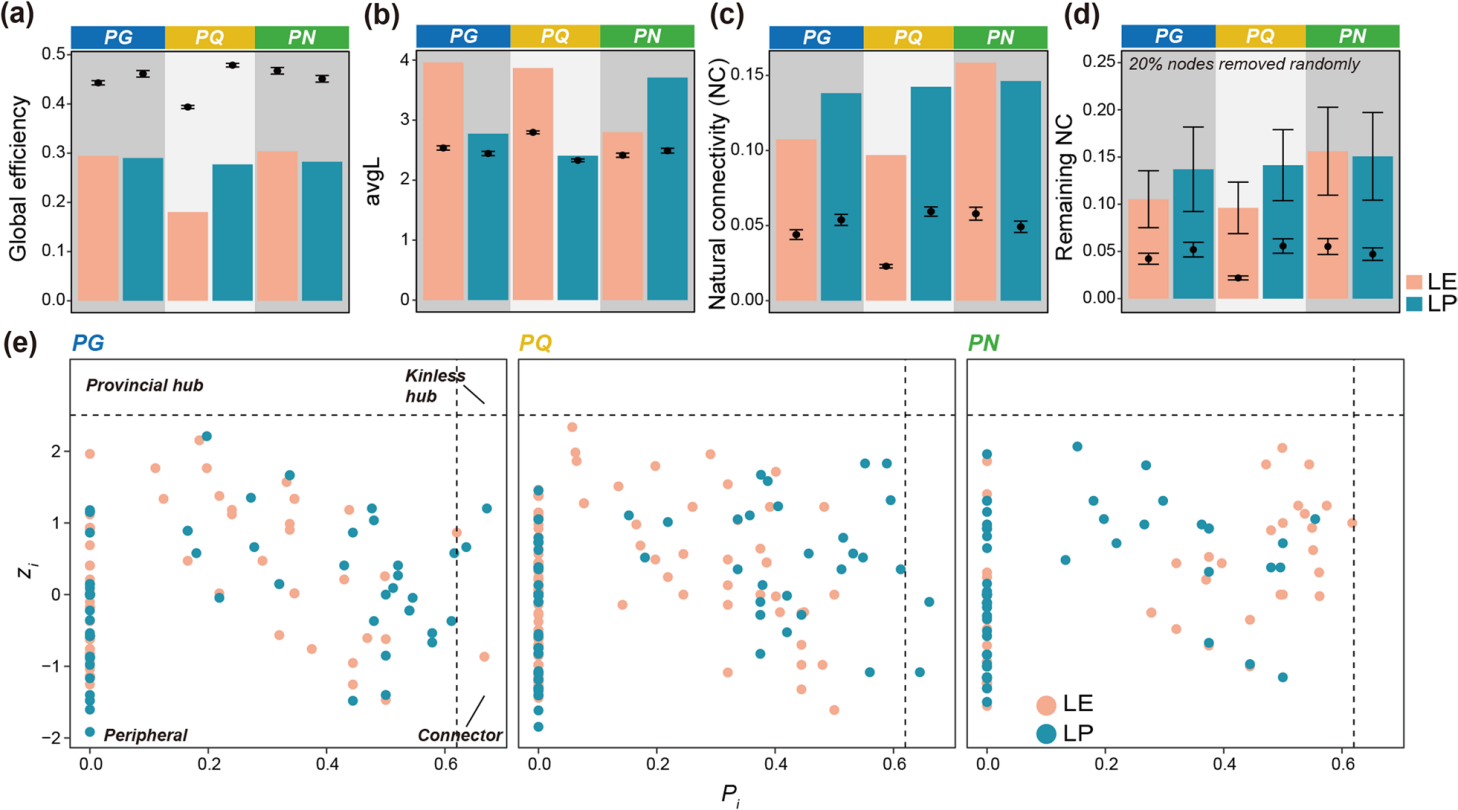
Comparison of network properties between observed and random graphs and the *z*_*i*_-*P*_*i*_ distribution of nodes. Global efficiency **a**, avgL (average shortest path length) **b**, natural connectivity of complete graph **c**, and remaining natural connectivity after 20% nodes removed randomly **d**. The bar plots represent the results of observed networks, while the error bar plots represent the mean and 1.96-fold of standard deviation. In **d**, the error bar of bar plot represents the standard deviation of 100 times random removal from observed network. **e** The *z*_*i*_-*P*_*i*_ distribution of nodes in the six MENs. The vertical lines were 0.62 and the horizontal lines were 2.5, which represent the classification threshold of node roles

Several complexity indices, including degree centralization, clustering coefficient, and modularity, were calculated for observed MENs and corresponding 100 random graphs. All these indices were significantly higher for true networks than random ones (higher than 1.96 standard deviations, *P* < 0.05), indicating the complex organization of phyllosphere mycobiomes (Additional file 1: Fig. S3). The homeostasis maintenance strategies of these MENs were further estimated. Compared with random graphs, the observed networks exhibited significantly lower global efficiency and higher avgL, which both indicated the low exchange efficiency among fungal nodes in *Panax* phyllosphere (Figs. 2a–b). In contrast, the comparison of natural connectivity between complete true and random networks indicated that the observed MENs were significantly more robust than random graphs (*P* < 0.05) (Fig. 2c). The remaining natural connectivity of MENs with 20%, 30%, and 40% of nodes being removed randomly were also significantly higher than those of random graphs (Fig. 2d**,** Additional file 1: Fig. S4). Additionally, a strong correlation of differences between observed and random natural connectivity with those between observed and random global efficiency was observed (Least square regression, *R*^*2*^ = 0.64, *P* = 0.05), which might represent a trade-off between robustness and efficiency in phyllosphere networks of *Panax* plants (Additional file 1: Fig. S5).

The *z*_*i*_-*P*_*i*_ framework was applied to identify hub nodes. Only few nodes, however, were categorized as connectors (*z*_*i*_ ≤ 2.5, *P*_*i*_ > 0.62) in all MENs, and no fungal taxa were identified as provincial hubs (*z*_*i*_ > 2.5, *P*_*i*_ < 0.62), let alone kinless hubs (*z*_*i*_ > 2.5, *P*_*i*_ > 0.62) (Fig. 2e). In together, these results strongly support our hypothesis ***H1***, meaning that the fungal MENs in *Panax* phyllosphere tend to apply the hub-independent high-robustness strategy to maintain homeostasis at the cost of network efficiency.

### Network modules contribute to shaping saponin profiles in *Panax* leaves

**Fig. 3:**
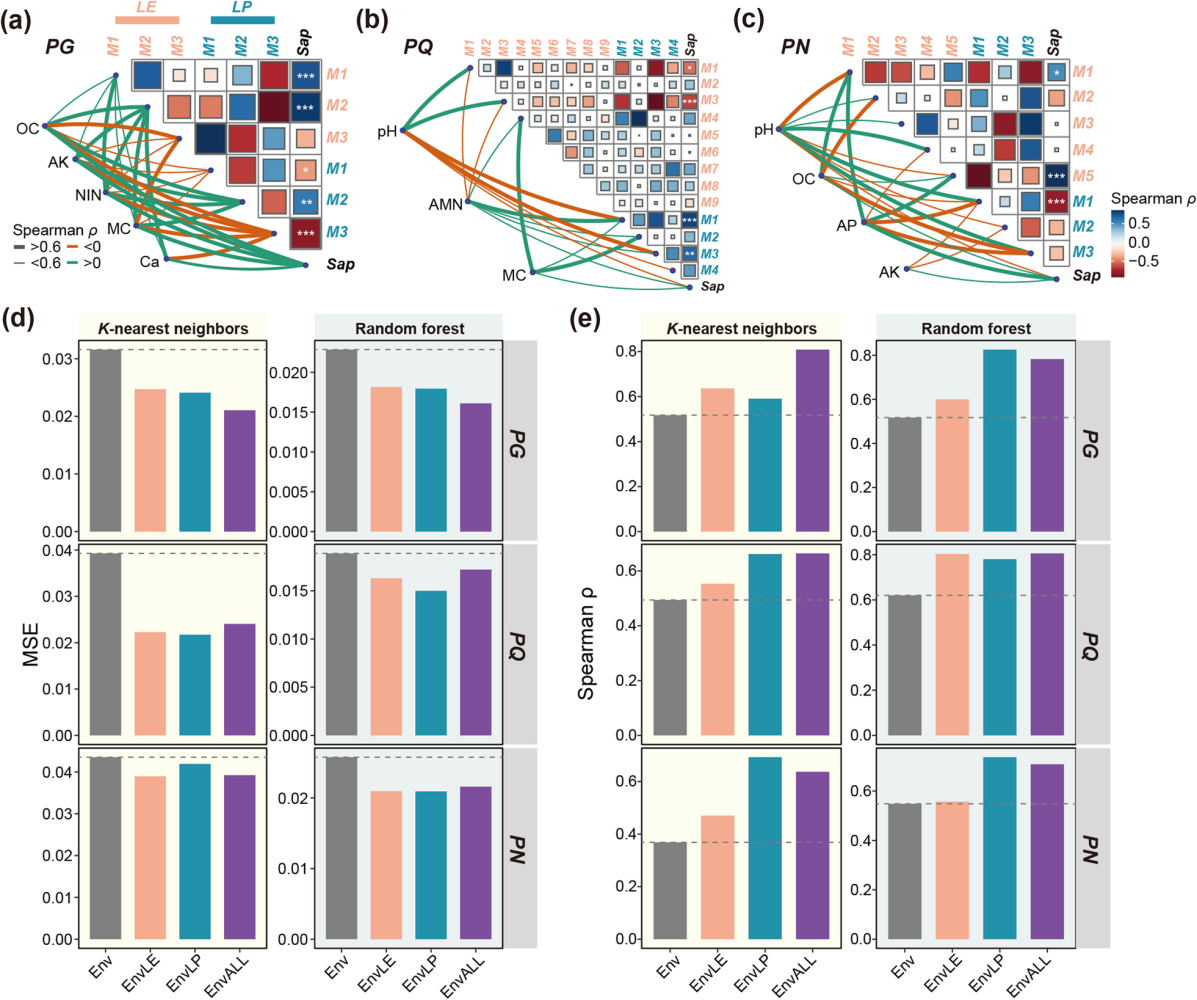
Contributions of fungal network modules to leaf saponin profiles. Correlations between eigengenes of modules, saponin profiles (Sap, the PC1 of leaf saponins), and environmental variables in PG **a**, PQ **b**, and PN **c**. Lines represent significant Spearman correlations (FDR < 0.05) between environmental factors and biotic factors. Heatmaps represent Spearman correlations among fungal modules and saponin profiles. *, FDR < 0.05; **, FDR < 0.01; ***, FDR < 0.001. MSE **d** and Spearman *ρ* **e** measures of four models constructed using two machine learning algorithms, respectively. Env represent models only containing environmental factors as features. Module eigengenes of LE and LP networks were further incorporated in EnvLE and EnvLP models, respectively. EnvALL models containing all environmental and module factors as features

In the six MENs, module eigengenes were calculated for 27 modules with 5 or more nodes to represent the overall variation pattern of all module members (Fig. 1a). Spearman correlation analysis indicated that the M1 and M2 in LE networks exhibited significant correlations (FDR < 0.05) with saponin variations in PG, while the candidate modules in LP network were M1, M2, and M3 (Fig. 3a). In PQ, LE modules 1 and 3, as well as LP modules 1 and 3 were significantly correlated with PC1 of leaf saponin profiles (Fig. 3b). In PN, the candidate modules were identified as M1 and M5 in LE, as well as M1 in LP (Fig. 3c). Correlations between saponin PC1 and environmental factors were also determined. Soil OC, AK, NIN, MC, and Ca were candidates (FDR < 0.05) potentially contributing to saponin variations in PG (Fig. 3a). In PQ, soil pH, AMN, and MC exhibited significant correlations with leaf saponin PC1 (Fig. 3b). Soil pH, OC, AP, and AK were the selected environmental factors in PN (Fig. 3c).

A machine learning-based strategy was applied to evaluate the contribution of fungal network modules to saponin variations in *Panax* leaves. In K-nearest neighbors regression, the mean squared error (MSE) and Spearman *ρ* both indicated that models incorporated with candidate fungal modules, no matter from LE or LP, performed better than models only containing candidate environmental features in all three *Panax* species (Fig. 3d–e). The random forest regression also supported the above observation (Fig. 3d–e). These results supported the critical roles of specific phyllosphere fungal modules in shaping saponin profiles of *Panax* leaves, providing evidence for our hypothesis ***H2***.

### 3.4 Positive regulation modules of total saponin accumulation were assembled by more deterministic ecological processes, comprising highly connected nodes and more plant-associated guilds

**Fig. 4:**
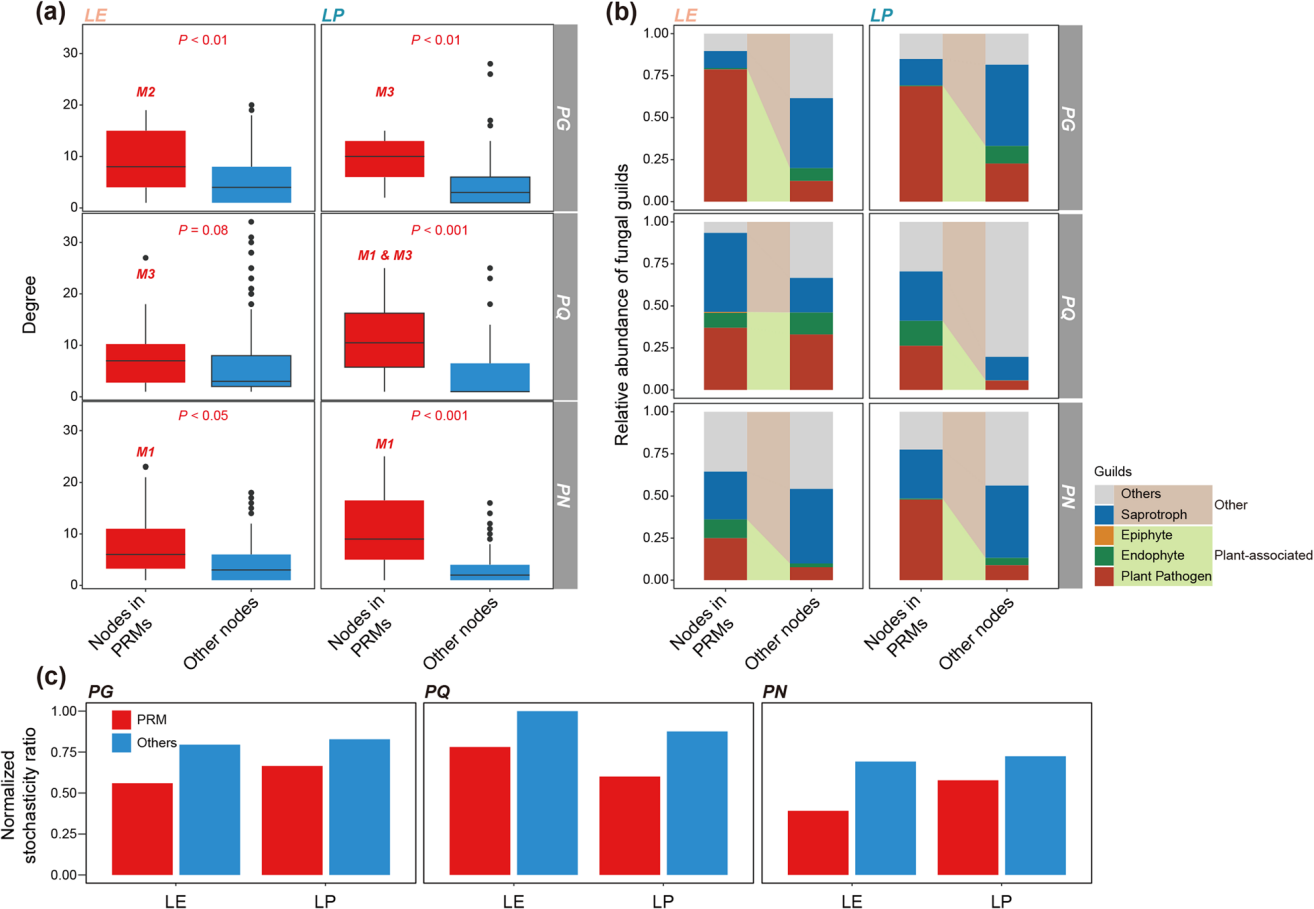
Comparison of positive regulation modules with other network members in terms of topological importance, guilds, and ecological assembly. **a** Comparison of degree between nodes in positive regulation modules (PRMs) and other nodes in the same network. *P* values were calculated using Wilcoxon rank-sum test. **b** Relative abundances of different functional guilds in PRMs and other nodes in the same network. Fungal guilds plant pathogen, endophyte, and epiphyte are considered as plant-associated guilds. **c** The relative contribution of stochastic processes to the assembly of PRM subcommunity and other network members. Red and blue bars represent RPM subcommunities and other nodes, respectively

Fungal ASVs in candidate modules identified above were further analysed for their correlations with contents of individual and total saponin (Additional file 1: Fig. S6). Modules with more than half of the number of positively correlated ASVs with total saponin content were identified as positive regulation modules (PRMs) of saponin accumulation, including M2 in LE and M3 in LP of PG, M3 in LE, M1 and M3 in LP of PQ, as well as M1 in LE and M1 in LP of PN (Additional file 1: Fig. S6). Members in these PRMs exhibited significantly (Wilcoxon rank sum test, *P* < 0.05) or nearly significantly (*P* = 0.08 for LE of PQ) higher degree than other nodes in the same MEN (Fig. 4a), indicating a more central position of these nodes in community structure. Besides, the proportions of plant-associated fungal guilds such as plant pathogen, endophyte, and epiphyte, were generally higher in PRMs compared to the proportions in other nodes, suggesting the tighter links between PRMs and *Panax* plant (Fig. 4b). The examination of ecological assembly mechanisms (NST) also supported the potentially tighter links between PRMs and host plant compared with other nodes, as the relative contributions of stochastic processes to the subcommunities assembly were higher for other nodes, indicating a more deterministic assembly of PRM members (Fig. 4c).

**Fig. 5:**
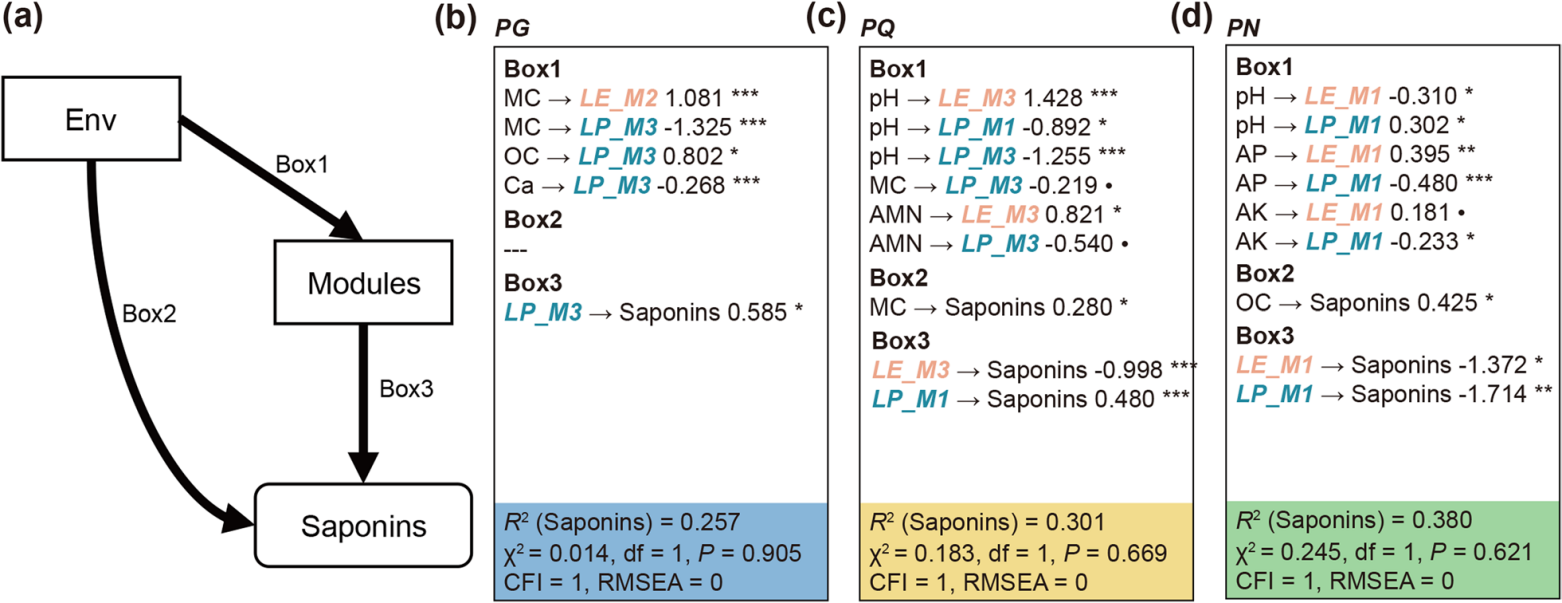
Structural equation model describing the direct effects of positive regulation modules on total saponin accumulation. **a** The prior model, on the basis of which the structural equation models were fitted. In the prior model, “Modules” indicated the eigengenes of positive regulation modules identified in each *Panax* species, and “Saponins” indicated the sum of contents of different saponins, representing total saponin accumulation in *Panax* leaves. The significant effect sizes calculated by structural equation models in PG **b**, PQ **c**, and PN **d** were displayed. Model statistics were shown in bottom of each box

The SEM was fitted to estimate whether there were significant and direct effects of PRMs on total saponin accumulation based on the prior model (Fig. 5a). The χ^2^ test, CFI and RMSEA statistics all supported the good fitting of models of three *Panax* species (Fig. 5b–d). In PG, the M3 in LP (Effect size = 0.585, *P* < 0.05) showed significant and direct effect on total saponin content (Fig. 5a). The M3 in LE (-0.998, *P* < 0.001) and M1 in LP (0.480, *P* < 0.001) were direct regulators on leaf total saponin in PQ (Fig. 5c). In PN, the M1 in LE (-1.372, *P* < 0.05) and M1 in LP (-1.714, *P* < 0.01) exhibited significant and direct effects on total saponin accumulation (Fig. 5d). Notably, the direct effects of environmental factors on total saponin content were rare and weak, highlighting the importance of fungal network modules in promoting leaf saponin accumulation (Fig. 5b–d). In general, these results indicated the topological importance of positive regulation modules, and emphasized their potentially tight interactions with plant host, as well as their direct effects on total saponin accumulation in *Panax* leaves.

### Specific and conservative positive regulation taxa of total saponin accumulation across *Panax* species

**Fig. 6:**
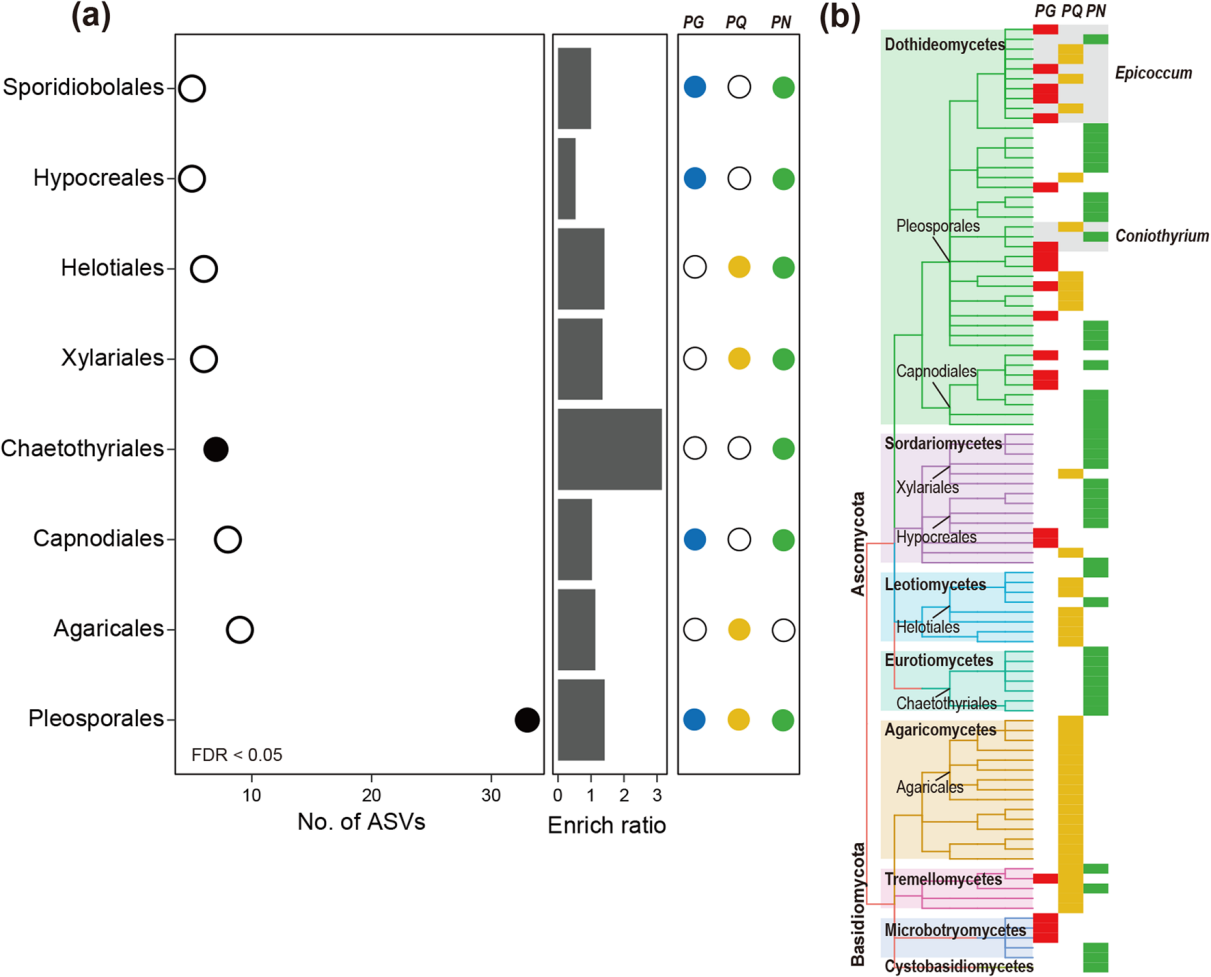
Order-level enrichment and taxonomic distribution of positive regulation taxa across three *Panax* species. **a** Order-level enrichment analysis of positive regulation taxa (PRT) across all six networks. Solid points represent the corresponding orders were significantly overrepresented by PRT (Fisher’s exact test, FDR < 0.05). **b** Taxonomic distribution of PRT and their absence/presence in the PRT group of each *Panax* species. The phylogenetic tree was constructed based on taxonomy, and clade colors represent different fungal classes

In SEM-validated positive regulation modules, nodes with significant and positive correlations with total saponin content were identified as positive regulation taxa (PRT) of leaf saponin accumulation (Additional file 1: Table S2). As there were strong collinearity between M2 in LE and M3 in LP of PG (Spearman *ρ* = − 0.92, *P* < 0.001; Additional file 1: Fig. S7), which might make the former be excluded from SEM, we also bring the M2 in LE into PRT analysis. Order-level enrichment analysis of these taxa from all six MENs showed that only two fungal orders were significantly overrepresented by PRT (Fisher’s exact test, FDR < 0.05), including Pleosporales and Chaetothyriales (Fig. 6a). In which, the Pleosporales was observed in networks of all three *Panax* species, while the Chaetothyriales only exhibited in MENs of PN (Fig. 6a). Additionally, all PRT were mapped on a taxonomy-based phylogenetic tree to estimate whether they were specific or conservative across *Panax* species. Almost all PRT present in PG networks exhibited relatives in PRT of PQ and PN (Fig. 6b). Obvious taxonomic specificity, however, could be observed for PQ and PN, respectively. For instance, PRT belonging to Chaetothyriales were specific to PN, and PRT from Agaricales only exhibited in PQ networks (Fig. 6b). Taxa of Helotiales presented in networks of both PQ and PN, while taxa of Microbotryomycetes were found in networks of both PG and PN. At genus level, only PRT belonging to *Epicoccum* and *Coniothyrium* could be found in MENs all three *Panax* species, indicating the conservatism of these taxa in improving leaf saponin accumulation across *Panax* species (Fig. 6b). In summary, these results revealed the specificity and conservatism of fungal taxa which potentially contributed to the accumulation of leaf total saponin across three *Panax* species.

## Discussion

In this study, we utilized microbial ecology network analysis to test two hypotheses regarding MEN organization strategy and the effects of network members on saponin metabolism in leaf-associated niches of three *Panax* species. Our findings revealed that the phyllosphere mycobiome employed a hub-independent structural robustness strategy at the cost of low efficiency. Moreover, certain modules in fungal networks contributed to the formation of leaf saponin profiles and the accumulation of leaf total saponin. The positive regulation modules of total saponin content located in more central positions of networks and displayed potentially tighter interactions with plant host than other nodes. Additionally, the positive regulation taxa of total saponin demonstrated specificity and conservatism across three *Panax* species.

### Highly robust but low-efficiency fungal networks in phyllosphere

Understanding how MEN was organized can help us reveal the maintenance strategies of mycobiome structural homeostasis, which can enhance our ability to manipulate microbial communities and is important for improving ecosystem services and plant performance [30, 69]. Many studies have revealed the small-world property (i.e., short path length between nodes) of ecological networks in bacterial communities, with highly connected hub nodes that provide a structural basis for efficient exchange of substances or information among community members [74, 77]. However, the organization and characteristics of phyllosphere mycobiome network is rarely studied. The present study showed that phyllosphere fungal MENs were highly robust but less efficiency than random graphs, implying an extreme homeostasis maintenance strategy of phyllosphere mycobiome. A previous study also found that fungal network structure was more stable than bacterial networks in response to drought stress [17]. The resource-poor environment in phyllosphere may be the driver of the extreme strategy employed by fungal communities. Compared to rhizosphere which is rich in minerals and plant-derive nutrients, the barren phyllosphere cannot afford fungi to quickly response to environmental disturbance in terms of energy demands [75]. In addition, stress like nutrition deficiency could drive the replacement of active taxa by slow-growing and stress-tolerant species which would also decrease the overall efficiency of networks [27].

Researches in grassland, alpine meadow, and agriculture ecosystems have emphasized the tight links between microbial network complexity and multiple ecosystem functions [79], [30, 74]. The highly complexity of fungal MENs observed in *Panax* phyllosphere thus provides basis for their important biological or ecological functions. Many studies of soil microbiomes have shown that ecosystem functions like biogeochemical cycling were mediated by hub nodes in MENs, which have key functional potential and are disproportionally important in structuring microbial communities [56]. The present study, nevertheless, indicated that there might not be such strong functional hubs in phyllosphere mycobiomes, in which members tended to tightly connect to each other in subgraphs. These subgraphs, rather than specific one or several nodes, may be the pivotal units for ecological or biological functions of phyllosphere mycobiomes [51]. Additionally, hub microorganisms have been recognized as the key for establishing desired microbiomes for sustainable agriculture [51], [63]. The hub-dependent mycobiome optimization strategy may face challenges in phyllosphere, as communities around a core may not be robust enough to persist in phyllosphere. Constructing and applying stable modules through synthetic community provides an alternative way to reshape phyllosphere mycobiome for improved plant performance and ecosystem services [75].

### Phyllosphere fungal modules contribute to saponin accumulation in *Panax* leaves and are tightly linked to host plant

Our analysis of fungal modules in LE and LP provided further validation for their important roles in shaping saponin profiles in *Panax* leaves, indicating that phyllosphere fungi may interact with plant host in the form of tightly connected clusters. Network modules as functional units of microorganisms have been widely reported in various ecosystems. For example, Liu et al. [75] found that modules in soil multitrophic networks could regulate the level of secondary metabolites in licorice root. Other researches in ecosystems such as sponge and human gut also suggested the functional importance of microbial modules [40, 67]. More importantly, positive regulation modules identified in the present study comprised a higher proportion of plant-associated fungi particularly potential plant pathogen, and exhibited more deterministic assembly processes. This finding is in line with our expectations, as saponins are a type of secondary metabolites closely related to plant-biotic factor interplay, and the plant–microbe war is a key driver of saponin evolution and biosynthesis [3, 24, 52]. In addition, the topological importance of members in PRMs indicated that these taxa not only mediated plant-mycobiome interactions but also within-mycobiome interplays. Actually, the importance in these two types of interactions is intrinsically related, as fungi tightly linked to plants can perceive and spread plant-associated information or materials, such as hormones and VOCs, more quickly than other nodes, thus occupying more central positions in MENs [71]. Previous studies in the model plant *Arabidopsis thaliana* also indicated that topologically important taxa in plant-associated microbial networks mediated interplays between the plant and microbial communities (van der Heijden and Hartmann, 2016). The significant and direct effects of most PRMs on total saponin accumulation were confirmed by SEM, further demonstrating the potential of these functional units to improve the accumulation of desired bioactive compounds in medicinal plant.

The specificity and conservatism of positive regulation taxa across three *Panax* species were identified on the basis of module members. Only fungi belonging to *Epicoccum* and *Coniothyrium* of Pleosporales were identified as PRT in all three *Panax* species. *Epicoccum* is a ubiquitous fungal genus. Many *Epicoccum* members have been found in niches associated with diverse plant species, some of which exhibit an invasion lifestyle [60]. *Coniothyrium* is also reported to be associated with the leaves of many plant species [46]. The conservative chemical response of *Panax* leaves to these fungi suggests that certain molecular patterns present in the two genera might be recognized by receptors shared by *Panax* species, ultimately leading to the up-regulation of saponin accumulation [43]. These fungi are thus useful for exploring conservative molecular mechanisms underlying the regulation of saponin biosynthesis in *Panax* genus. Additionally, many *Panax* species-specific PRT were also found diverse taxonomic groups, which may reflect the differences in the fungal species pool exposed to three *Panax* species caused by environmental filtering and/or dispersal limitation [35]. Furthermore, new types of plant-fungi interactions driven by plant divergence and evolution may also contribute to this specificity [57, 58]. Consequently, the positive regulation taxa and the modules formed by them may serve as the foundation for engineering mycobiome to improving leaf saponin accumulation in *Panax* and help us understand the molecular and chemical mechanisms of plant-fungi interactions.

## Conclusions

In summary, this study revealed the high-robustness but low-efficiency network organization strategy of phyllosphere mycobiomes and validated the contributions of network modules to saponin profiles and total saponin content in *Panax* leaves. These findings provide foundations for module-based phyllosphere mycobiomes engineer aiming at the promotion of bioactive secondary metabolite accumulation. The specificity and conservatism of positive regulation fungal taxa across three *Panax* species enhance our understanding of plant-fungi interactions and coevolution associated with secondary metabolism.

## Supplementary Information

**Additional file 1**. Supplementary figures and tables.

## Data Availability

The datasets generated and/or analysed during the current study are available in the National Genomics Data Center (https://ngdc.cncb.ac.cn) under the BioProject PRJCA007643. *R* codes used for statistical analyses are available at https://github.com/githubzgz.

## Abbreviations

AK: Available potassium
AMN: Ammonium nitrogen
AP: Available phosphorus
AS: Available sulfur
ASV: Amplicon sequence variants
ECa: Exchangeable calcium
EMg: Exchangeable magnesium
GOE: Gaussian orthogonal ensemble
LTED: Link test for environmental filtering or dispersal limitation
MC: Moisture content
MEN: Microbial ecological network
NIN: Nitrate nitrogen
NST: Normalized stochasticity ratio
OC: Organic carbon
PG: *Panax ginseng*
PQ: *Panax quinquefolium*
PN: *Panax notoginseng*
PRM: Positive regulation module of total saponin accumulation
PRT: Positive regulation taxa of total saponin accumulation
TN: Total nitrogen
RMT: Random matrix theory
SEM: Structural equation module
