# Supplementary material for: Modules in robust but low-efficiency phyllosphere fungal networks drive saponin accumulation in leaves of different Panax species
Source: Environ Microbiome. 2023 Jul 12;18:57. doi: 10.1186/s40793-023-00516-7 (PMC10337071; doi:10.1186/s40793-023-00516-7)
Supplement: Supplementary file 1 — Additional file 1. Supplementary figures and tables. [file 40793_2023_516_MOESM1_ESM.docx]

*Supplementary materials for*

**Modules in robust but low-efficiency phyllosphere fungal networks drive saponin accumulation in leaves of different *Panax* species**

Guozhuang Zhang^1^, Liping Shi^1^, Liu Congsheng^2^, Renjun Mao^3^, Huang Zhixin^2^, Xiuye Wei^1^, Lixuan Wu^2^, Zheng Yuqing^2^, Guangfei Wei^1^, Jia Xu^1^, Shuangrong Gao^1^, Shilin Chen^1, 4^, Linlin Dong^1^

*^1^ Key Laboratory of Beijing for Identification and Safety Evaluation of Chinese Medicine, Institute of Chinese Materia Medica, China Academy of Chinese Medical Sciences, Beijing, 100700, China*

*^2^ Zhangzhou Pianzihuang Pharmaceutical Co., Ltd., Fujian, 363099, China*

*^3^ School of Life Sciences, Yan’ an University, Yan’ an, 716000, China*

*^4^ Institute of Herbgenomics, Chengdu University of Traditional Chinese Medicine, Chengdu, Sichuan, China*

**Contents**

**Fig. S1** Sampling location and *Panax* cultivation.

**Fig. S2** Distribution and power-law fit of node degree of phyllosphere networks.

**Fig. S3** Complexity indices of phyllosphere networks and corresponding random graphs.

**Fig. S4** Natural connectivity calculated from networks with different proportions of nodes randomly removed.

**Fig. S5** Correlations between differences of global efficiency between observed and random graphs and differences of natural connectivity between observed and random graphs.

**Fig. S6** Correlations between members of candidate modules and saponin contents.

**Fig. S7** Correlations between eigengenes of M3 in LP of PG and M2 in LE of PG.

**Table S1** Basic topological properties of fungal MENs in *Panax* phyllosphere.

**Table S2** Taxonomic distribution of positive regulation taxa in SEM-validated positive regulation modules across three *Panax* species.

**Fig. S1** Sampling location and *Panax* cultivation. (a) Sampling locations of three *Panax* species and corresponding mean annual temperature; (b) Picture of cultivated *Panax* covered by sunshade.

**
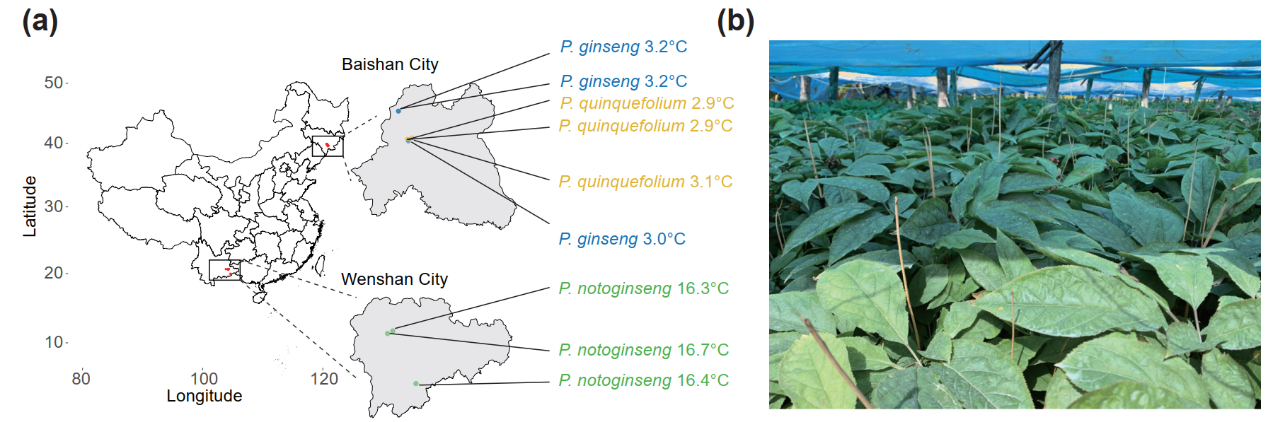
**

**Fig. S2** Distribution and power-law fit of node degree of phyllosphere networks. Dash lines represent median value of node degree. P values were calculated based on Kolmogorov-Smirnov test on node degree higher than median. N indicated number of nodes with degree higher than median.


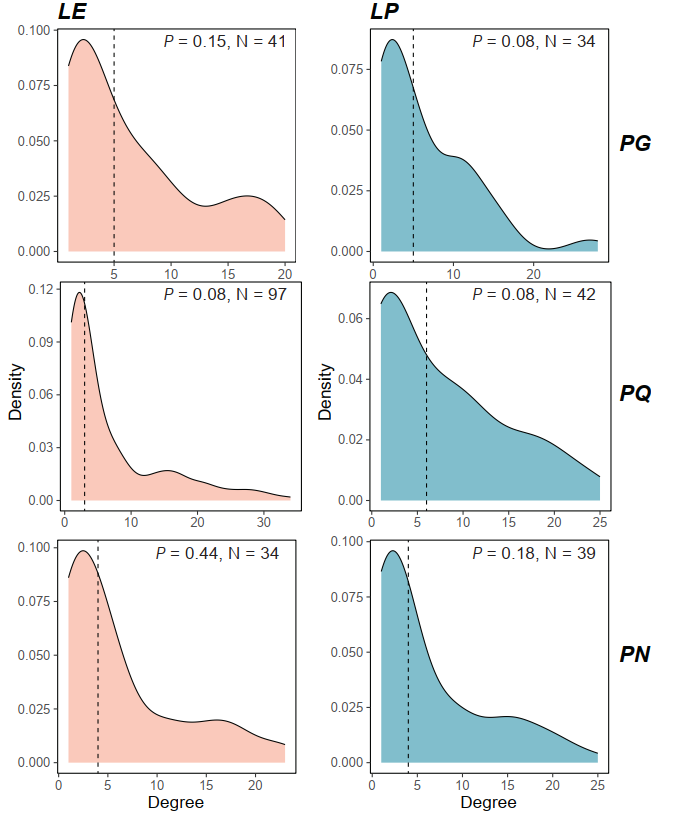


**Fig. S3** Complexity indices of phyllosphere networks and corresponding random graphs. Three complexity indices, including degree centralization, clustering coefficient, and modularity were calculated for observed MENs and 100 random graphs. Bar plots represented the value of observed MENs. Black dots indicated the mean value of random graphs, while error bars represent 1.96 standard deviations.


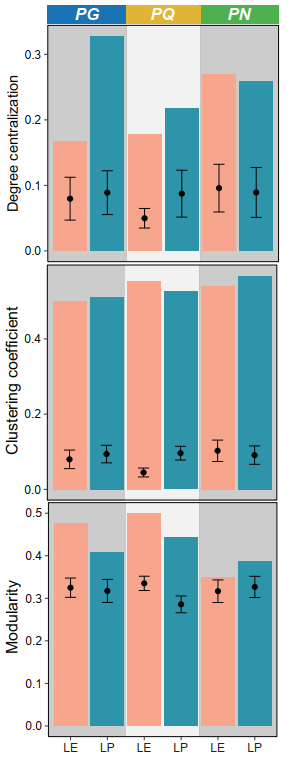


**Fig. S4** Natural connectivity calculated from networks with different proportions of nodes randomly removed. Bar plots and error bars represented the mean and 1.96 standard deviations of observed MENs, while black dots and error bars indicated the mean and 1.96 standard deviations of random graphs.


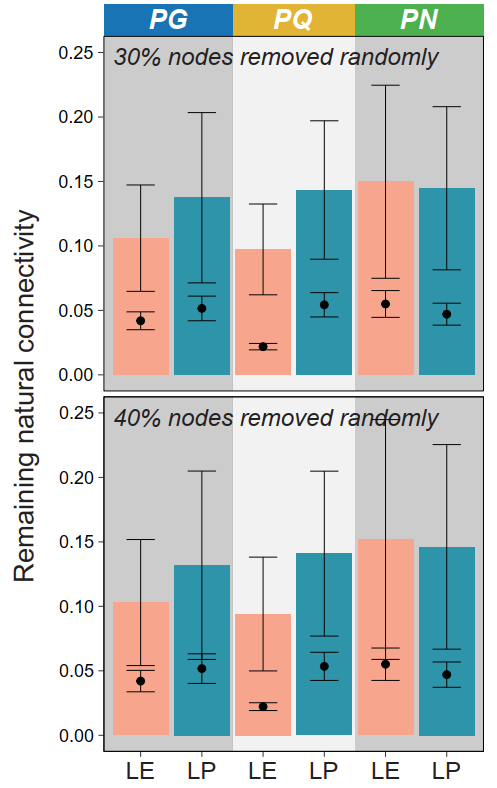


**Fig. S5** Correlations between differences of global efficiency between observed and random graphs and differences of natural connectivity between observed and random graphs. Differences were represented using the number of standard deviations. *R^2^* and *P* value were statistics of least square regression.


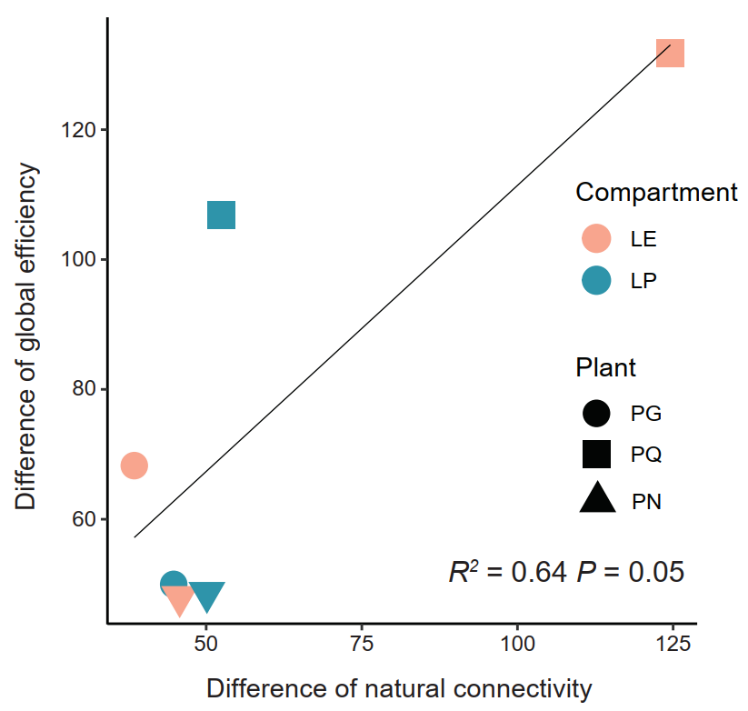


**Fig. S6** Correlations between members of candidate modules and saponin contents. Colored tiles represent significant Spearman correlations (FDR < 0.05). Rows were ASVs in different modules and columns were different types of saponins and the total saponin content. Candidate modules indicated those with significant correlations with saponin profiles (PC1).


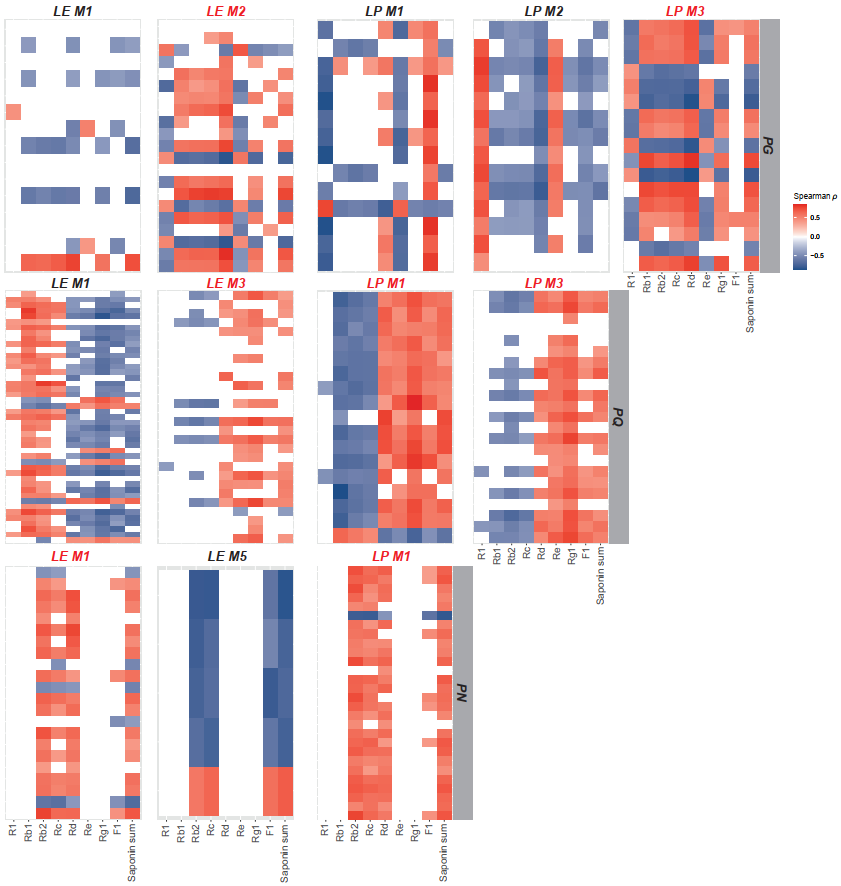


**Fig. S7** Correlations between eigengenes of M3 in LP of PG and M2 in LE of PG.


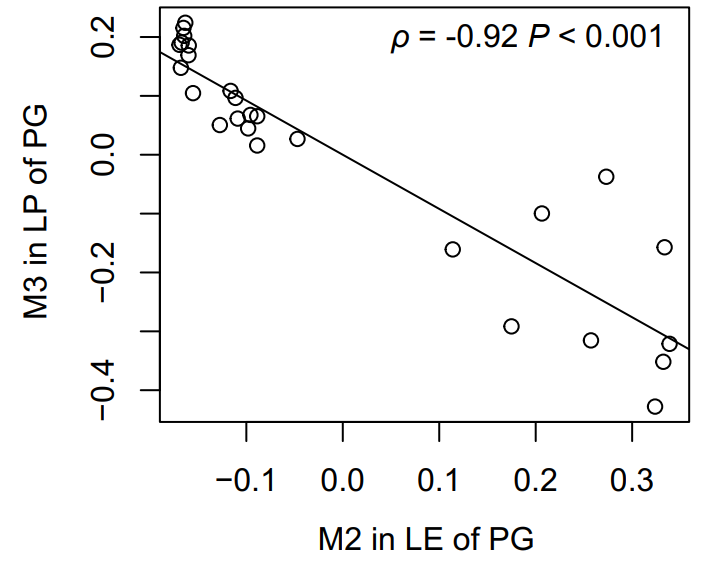


**Table S1** Basic topological properties of fungal MENs in *Panax* phyllosphere.

| Plant | Compartment | N | E | GD | AvgL | CC | GE | NC | M | St |
| --- | --- | --- | --- | --- | --- | --- | --- | --- | --- | --- |
| PG | LE | 82 | 267 | 0.080 | 3.962 | 0.502 | 0.295 | 0.107 | 0.476 | 0.6242 |
| PG | LP | 67 | 213 | 0.096 | 2.774 | 0.511 | 0.290 | 0.138 | 0.408 | 0.7636 |
| PQ | LE | 153 | 530 | 0.046 | 3.869 | 0.553 | 0.180 | 0.097 | 0.500 | 0.6667 |
| PQ | LP | 80 | 313 | 0.099 | 2.407 | 0.526 | 0.277 | 0.142 | 0.442 | 0.7858 |
| PN | LE | 63 | 199 | 0.102 | 2.798 | 0.541 | 0.304 | 0.159 | 0.349 | 0.7159 |
| PN | LP | 73 | 233 | 0.089 | 3.709 | 0.568 | 0.283 | 0.146 | 0.386 | 0.7576 |

N: Number of nodes;

E: Number of edges;

GD: Graph density;

AvgL: Average shortest path length;

CC: Clustering coefficient;

GE: Global efficiency;

NC: Natural connectivity;

M: Modularity (fast greedy algorithm);

St: Correlation threshold selected based on random matrix theory.

**Table S2** Taxonomic distribution of positive regulation taxa in SEM-validated positive regulation modules across three *Panax* species.

| Phylum | Class | Order | Family | Genus | Plant | Compartment | Module |
| --- | --- | --- | --- | --- | --- | --- | --- |
| Ascomycota | Dothideomycetes | Capnodiales | Cladosporiaceae | Cladosporium | Gin | LE | M2 |
| Ascomycota | Dothideomycetes | Pleosporales | Coniothyriaceae | Coniothyrium | Gin | LE | M2 |
| Ascomycota | Dothideomycetes | Pleosporales | Didymellaceae | Epicoccum | Gin | LE | M2 |
| Ascomycota | Dothideomycetes | Pleosporales | Didymellaceae | Epicoccum | Gin | LE | M2 |
| Ascomycota | Dothideomycetes | Pleosporales | Didymellaceae | Epicoccum | Gin | LE | M2 |
| Ascomycota | Dothideomycetes | Pleosporales | Massarinaceae | Stagonospora | Gin | LE | M2 |
| Ascomycota | Dothideomycetes | Pleosporales | norank | Dokmaia | Gin | LE | M2 |
| Ascomycota | Dothideomycetes | Pleosporales | Pleosporaceae | Hendersonia | Gin | LE | M2 |
| Basidiomycota | Microbotryomycetes | Sporidiobolales | Sporidiobolaceae | Sporidiobolus | Gin | LE | M2 |
| Ascomycota | Dothideomycetes | Capnodiales | Cladosporiaceae | Cladosporium | Gin | LP | M3 |
| Ascomycota | Dothideomycetes | Capnodiales | Cladosporiaceae | Cladosporium | Gin | LP | M3 |
| Ascomycota | Sordariomycetes | Hypocreales | Hypocreaceae | Hypomyces | Gin | LP | M3 |
| Ascomycota | Dothideomycetes | Pleosporales | Didymellaceae | Epicoccum | Gin | LP | M3 |
| Ascomycota | Dothideomycetes | Pleosporales | Didymellaceae | Epicoccum | Gin | LP | M3 |
| Ascomycota | Dothideomycetes | Pleosporales | Lophiostomataceae | Biappendiculispora | Gin | LP | M3 |
| Ascomycota | Dothideomycetes | Pleosporales | Massarinaceae | Stagonospora | Gin | LP | M3 |
| Basidiomycota | Microbotryomycetes | Sporidiobolales | Sporidiobolaceae | Sporidiobolus | Gin | LP | M3 |
| Basidiomycota | Microbotryomycetes | Sporidiobolales | Sporidiobolaceae | Sporidiobolus | Gin | LP | M3 |
| Basidiomycota | Tremellomycetes | Tremellales | Bulleribasidiaceae | Vishniacozyma | Gin | LP | M3 |
| Ascomycota | Sordariomycetes | Trichosphaeriales | Trichosphaeriaceae | Nigrospora | Gin | LP | M3 |
| Ascomycota | Dothideomycetes | Capnodiales | Cladosporiaceae | Cladosporium | Not | LE | M1 |
| Ascomycota | Dothideomycetes | Capnodiales | Teratosphaeriaceae | Catenulostroma | Not | LE | M1 |
| Ascomycota | Eurotiomycetes | Chaetothyriales | Herpotrichiellaceae | Rhinocladiella | Not | LE | M1 |
| Ascomycota | Eurotiomycetes | Chaetothyriales | norank | Strelitziana | Not | LE | M1 |
| Ascomycota | Eurotiomycetes | Chaetothyriales | norank | Strelitziana | Not | LE | M1 |
| Ascomycota | Eurotiomycetes | Chaetothyriales | norank | Strelitziana | Not | LE | M1 |
| Ascomycota | Eurotiomycetes | Chaetothyriales | norank | Strelitziana | Not | LE | M1 |
| Ascomycota | Sordariomycetes | Glomerellales | Glomerellaceae | Colletotrichum | Not | LE | M1 |
| Ascomycota | Leotiomycetes | Helotiales | Sclerotiniaceae | Botrytis | Not | LE | M1 |
| Ascomycota | Sordariomycetes | Hypocreales | Sarocladiaceae | Sarocladium | Not | LE | M1 |
| Ascomycota | Dothideomycetes | Pleosporales | Didymosphaeriaceae | Pseudopithomyces | Not | LE | M1 |
| Ascomycota | Dothideomycetes | Pleosporales | Phaeosphaeriaceae | Phaeosphaeriopsis | Not | LE | M1 |
| Ascomycota | Dothideomycetes | Pleosporales | Phaeosphaeriaceae | Setophoma | Not | LE | M1 |
| Ascomycota | Dothideomycetes | Pleosporales | Pleosporaceae | Bipolaris | Not | LE | M1 |
| Basidiomycota | Microbotryomycetes | Sporidiobolales | Sporidiobolaceae | Sporidiobolus | Not | LE | M1 |
| Basidiomycota | Tremellomycetes | Tremellales | Bulleribasidiaceae | Bulleribasidium | Not | LE | M1 |
| Ascomycota | Sordariomycetes | Xylariales | Microdochiaceae | Microdochium | Not | LE | M1 |
| Ascomycota | Dothideomycetes | Capnodiales | Cladosporiaceae | Rachicladosporium | Not | LP | M1 |
| Ascomycota | Dothideomycetes | Capnodiales | Cladosporiaceae | Rachicladosporium | Not | LP | M1 |
| Ascomycota | Dothideomycetes | Capnodiales | Mycosphaerellaceae | Ragnhildiana | Not | LP | M1 |
| Ascomycota | Eurotiomycetes | Chaetothyriales | Herpotrichiellaceae | Rhinocladiella | Not | LP | M1 |
| Ascomycota | Eurotiomycetes | Chaetothyriales | norank | Strelitziana | Not | LP | M1 |
| Basidiomycota | Cystobasidiomycetes | Erythrobasidiales | Erythrobasidiaceae | Erythrobasidium | Not | LP | M1 |
| Ascomycota | Leotiomycetes | Helotiales | Sclerotiniaceae | Botrytis | Not | LP | M1 |
| Ascomycota | Sordariomycetes | Hypocreales | Nectriaceae | Fusarium | Not | LP | M1 |
| Ascomycota | Sordariomycetes | Hypocreales | Sarocladiaceae | Sarocladium | Not | LP | M1 |
| Ascomycota | Sordariomycetes | Hypocreales | Stachybotryaceae | Myrothecium | Not | LP | M1 |
| Ascomycota | Dothideomycetes | Pleosporales | Coniothyriaceae | Coniothyrium | Not | LP | M1 |
| Ascomycota | Dothideomycetes | Pleosporales | Corynesporascaceae | Corynespora | Not | LP | M1 |
| Ascomycota | Dothideomycetes | Pleosporales | Didymellaceae | Didymella | Not | LP | M1 |
| Ascomycota | Dothideomycetes | Pleosporales | Didymellaceae | Epicoccum | Not | LP | M1 |
| Ascomycota | Dothideomycetes | Pleosporales | Phaeosphaeriaceae | Phaeosphaeriopsis | Not | LP | M1 |
| Ascomycota | Dothideomycetes | Pleosporales | Pleosporaceae | Bipolaris | Not | LP | M1 |
| Ascomycota | Dothideomycetes | Pleosporales | Pleosporaceae | Bipolaris | Not | LP | M1 |
| Ascomycota | Dothideomycetes | Pleosporales | Pleosporaceae | Curvularia | Not | LP | M1 |
| Ascomycota | Dothideomycetes | Pleosporales | Pyrenochaetopsidaceae | Pyrenochaetopsis | Not | LP | M1 |
| Basidiomycota | Microbotryomycetes | Sporidiobolales | Sporidiobolaceae | Sporidiobolus | Not | LP | M1 |
| Basidiomycota | Tremellomycetes | Tremellales | Bulleribasidiaceae | Vishniacozyma | Not | LP | M1 |
| Ascomycota | Sordariomycetes | Xylariales | Apiosporaceae | Arthrinium | Not | LP | M1 |
| Ascomycota | Sordariomycetes | Xylariales | Microdochiaceae | Microdochium | Not | LP | M1 |
| Ascomycota | Sordariomycetes | Xylariales | Microdochiaceae | Microdochium | Not | LP | M1 |
| Ascomycota | Sordariomycetes | Xylariales | Sporocadaceae | Neopestalotiopsis | Not | LP | M1 |
| Basidiomycota | Agaricomycetes | Agaricales | Entolomataceae | Entoloma | Qui | LP | M1 |
| Basidiomycota | Agaricomycetes | Agaricales | Physalacriaceae | Armillaria | Qui | LP | M1 |
| Ascomycota | Leotiomycetes | Erysiphales | Erysiphaceae | Golovinomyces | Qui | LP | M1 |
| Basidiomycota | Tremellomycetes | Filobasidiales | Filobasidiaceae | Filobasidium | Qui | LP | M1 |
| Ascomycota | Leotiomycetes | Helotiales | Sclerotiniaceae | Botrytis | Qui | LP | M1 |
| Ascomycota | Leotiomycetes | Helotiales | Sclerotiniaceae | Botrytis | Qui | LP | M1 |
| Ascomycota | Sordariomycetes | Microascales | Microascaceae | Scopulariopsis | Qui | LP | M1 |
| Ascomycota | Dothideomycetes | Pleosporales | Coniothyriaceae | Coniothyrium | Qui | LP | M1 |
| Ascomycota | Dothideomycetes | Pleosporales | Didymellaceae | Epicoccum | Qui | LP | M1 |
| Ascomycota | Dothideomycetes | Pleosporales | Didymellaceae | Epicoccum | Qui | LP | M1 |
| Ascomycota | Dothideomycetes | Pleosporales | Didymellaceae | Epicoccum | Qui | LP | M1 |
| Ascomycota | Dothideomycetes | Pleosporales | Periconiaceae | Periconia | Qui | LP | M1 |
| Ascomycota | Dothideomycetes | Pleosporales | Periconiaceae | Periconia | Qui | LP | M1 |
| Ascomycota | Dothideomycetes | Pleosporales | Pleosporaceae | Exserohilum | Qui | LP | M1 |
| Basidiomycota | Agaricomycetes | Russulales | Bondarzewiaceae | Heterobasidion | Qui | LP | M1 |
| Ascomycota | Sordariomycetes | Xylariales | Xylariaceae | Xylaria | Qui | LP | M1 |
| Basidiomycota | Agaricomycetes | Agaricales | Lyophyllaceae | Ossicaulis | Qui | LE | M3 |
| Basidiomycota | Agaricomycetes | Agaricales | Lyophyllaceae | Tephrocybe | Qui | LE | M3 |
| Basidiomycota | Agaricomycetes | Agaricales | Physalacriaceae | Armillaria | Qui | LE | M3 |
| Basidiomycota | Agaricomycetes | Agaricales | Physalacriaceae | Armillaria | Qui | LE | M3 |
| Basidiomycota | Agaricomycetes | Agaricales | Physalacriaceae | Cylindrobasidium | Qui | LE | M3 |
| Basidiomycota | Agaricomycetes | Agaricales | Tricholomataceae | Collybia | Qui | LE | M3 |
| Basidiomycota | Agaricomycetes | Agaricales | Tricholomataceae | Lepista | Qui | LE | M3 |
| Basidiomycota | Tremellomycetes | Cystofilobasidiales | Mrakiaceae | Itersonilia | Qui | LE | M3 |
| Ascomycota | Leotiomycetes | Erysiphales | Erysiphaceae | Phyllactinia | Qui | LE | M3 |
| Ascomycota | Leotiomycetes | Helotiales | Cenangiaceae | Heyderia | Qui | LE | M3 |
| Ascomycota | Leotiomycetes | Helotiales | Helotiaceae | Hymenoscyphus | Qui | LE | M3 |
| Ascomycota | Dothideomycetes | Pleosporales | Didymellaceae | Epicoccum | Qui | LE | M3 |
| Ascomycota | Dothideomycetes | Pleosporales | Lophiostomataceae | Lophiostoma | Qui | LE | M3 |
| Basidiomycota | Agaricomycetes | Polyporales | Polyporaceae | Lenzites | Qui | LE | M3 |
| Basidiomycota | Agaricomycetes | Polyporales | Polyporaceae | Trametes | Qui | LE | M3 |
| Basidiomycota | Agaricomycetes | Polyporales | Polyporaceae | Trametes | Qui | LE | M3 |
| Basidiomycota | Agaricomycetes | Russulales | Peniophoraceae | Peniophora | Qui | LE | M3 |
| Basidiomycota | Agaricomycetes | Russulales | Peniophoraceae | Peniophora | Qui | LE | M3 |
